# Stage-Dependent Expression of Protein Gene Product 9.5 in Donkey Testes

**DOI:** 10.3390/ani10112169

**Published:** 2020-11-20

**Authors:** Yeonju Choi, Youngwook Jung, Seongmin Kim, Junyoung Kim, Heejun Jung, Minjung Yoon

**Affiliations:** 1 Department of Animal Science and Biotechnology, Kyungpook National University, Sangju 37224, Korea; yjchoi2031@gmail.com (Y.C.); dyddnr02070@naver.com (Y.J.); kimhandles@gmail.com (S.K.); cpm_911@naver.com (J.K.); hjjung1019@gmail.com (H.J.); 2 Department of Horse, Companion, and Wild Animal Science, Kyungpook National University, Sangju 37224, Korea

**Keywords:** PGP9.5, donkey, testes, spermatogenesis

## Abstract

**Simple Summary:**

Spermatogenesis and steroidogenesis are key functions of the testes. Molecular markers that identify each stage of germ cells and Leydig cells can identify and isolate specific germ or Leydig cells. Protein gene product (PGP)9.5 is observed in neuroendocrine cells and tumors; it is also used for the immunohistochemical detection of spermatogonial stem cells (SSCs) in various species of animals. It was found that the immunolabeling of PGP9.5 in testicular tissue was not observed in the seminiferous tubules in the pre-pubertal stage. However, in the post-pubertal stage, spermatogonia were immunolabeled with PGP9.5. Interestingly, some Leydig cells were immunolabeled with PGP9.5 in both pre- and post-pubertal stages. This study reflects that the PGP9.5 antibody can be used as a tool to identify and isolate spermatogonia from seminiferous tubules in the post-pubertal stage of donkey testes.

**Abstract:**

Molecular markers can be used to identify and isolate specific developmental stages of germ cells and Leydig cells. Protein gene product (PGP)9.5 expression in spermatogonia and Leydig cells has been reported in several species. The stages of spermatogonia and Leydig cells expressing PGP9.5 vary depending on the species and reproductive stages. Thus, the objectives of this study were (1) to identify the localization of PGP9.5 in donkey testicular cells, and (2) to compare the expression patterns of PGP9.5 in donkey testicular cells between pre- and post-pubertal stages. Testes samples were collected following the routine field castration of six donkeys. Western blotting was performed to verify the cross-reactivity of the rabbit anti-human PGP9.5 antibody to donkey testes. Immunofluorescence was performed to investigate the expression pattern of PGP9.5 in testicular tissues at different reproductive stages. In Western blotting, the protein band of the PGP9.5 antibody appeared at approximately 27 kDa, whereas the band was not observed in the negative control treated with normal mouse IgG. In the pre-pubertal stage, the expression of deleted in azoospermia-like (DAZL) was found in some spermatogonia in pre-pubertal testicular tissues. However, the immunolabeling of PGP9.5 in testicular tissue was not observed in the seminiferous tubules. In stages 1 and 2, spermatogonia were immunolabeled with either PGP9.5 or DAZL. In contrast, PGP9.5 and DAZL were co-immunolabeled in some of the spermatogonia in stages 3 to 8. Interestingly, some Leydig cells were immunolabeled with PGP9.5 in both pre- and post-pubertal stages. In conclusion, the PGP9.5 antibody can be used as a tool to identify and isolate spermatogonia from seminiferous tubules.

## 1. Introduction

Spermatogenesis and steroidogenesis are key functions of the testes. Molecular markers that identify each stage of germ cells and Leydig cells can identify and isolate specific germ or Leydig cells. Using tools such as molecular markers, biological processes for the proliferation and differentiation of germ and Leydig cells can be explored. These markers can also be applied to develop advanced assisted reproductive techniques such as spermatogonial stem cell (SSC) or Leydig cell transplantation and diagnose subfertility or infertility in donkeys.

Previous studies have examined molecular markers for germ cells. Glial cell-derived neurotrophic factor family receptor alpha-1, promyelocytic leukemia zinc finger, and colony-stimulating factor 1 receptor were found to be markers for SSCs [1]. In a previous study, our laboratory reported that a co-immunolabeling system with undifferentiated embryonic cell transcription factor 1 (UTF1) and deleted in azoospermia-like (DAZL) could be used to identify undifferentiated (UTF1 only), differentiating (UTF1 and DAZL), or differentiated spermatogonia (DAZL only) [2]. Thus, the ability to more efficiently detect more molecular markers that can identify various stages of germ and Leydig cells would be beneficial.

Protein gene product (PGP)9.5, also known as ubiquitin C-terminal hydrolase L1 (UCHL1), was initially found to be expressed in neuroendocrine cells and tumors [3]. The function of PGP9.5 is to increase the available pool of ubiquitin that can attach to the proteins which are about to be degraded by the proteasome [4]. The expression of PGP9.5 was also reported in reproductive systems such as the testes in several animals, which include mice [5], cattle [6], pigs [7], sheep [8], and goats [9]. The expression of PGP9.5 was detected in spermatogonia and Leydig cells, but the immunolabeling pattern varies depending on the species. In addition, the PGP9.5 was immunolabeled in a reproductive stage-dependent manner. Thus, to confirm the localization of PGP9.5 in testes, the expression patterns of the marker must be tested in each species at different reproductive stages.

We recently reported that PGP9.5 was expressed in the germ cells adjacent to the seminiferous tubule membrane and cytoplasmic area of Leydig cells in stallions [10]. Based on the results of our study and other previous studies, we hypothesized that PGP9.5 is expressed in donkey testes, and it can be used as a marker for specific stages of germ cells and Leydig cells.

Thus, the objectives of this study were (1) to identify the localization of PGP9.5 in donkey testicular cells, and (2) to compare the expression patterns of PGP9.5 in donkey testicular cells between pre- and post-pubertal stages.

## 2. Materials and Methods

### 2.1. Testicular Sample Preparation

Testes were collected following the routine field castration of six donkeys from a private farm in Icheon, South Korea. The reproductive stages of donkeys were categorized based on the morphological characteristics of the cross sections of seminiferous tubules within donkey testes. Following castration, the testes were directly transported to the laboratory in a 4 °C icebox. The testes samples were then cut into small pieces (approximately 1 cm^3^), and the tissues were treated with paraformaldehyde for at least 24 h at room temperature. During the dehydration process, the sample tissues were immersed in phased ethanol concentrations of 25%, 50%, 70%, 80%, 90%, and 100%. Then, the tissues were embedded in paraffin blocks, and the tissue pieces (1 mg each) were snap-frozen in liquid nitrogen and stored at −80 °C for further Western blotting.

### 2.2. Western Blotting

Western blotting was performed to verify the cross-reactivity of rabbit anti-human PGP9.5 (7863-2004, Bio-Rad, Hercules, CA, USA) to the PGP9.5 present in donkey testes according to the previously reported protocol with minor modifications [2,11]. The testicular samples, which had been stored at −80 °C, were thawed at room temperature and homogenized using a Polytron PT 1200 CL homogenizer (Kinematica AG, Littau, Lucerne, Switzerland). Sample preparation buffer (0.5 M Tris-HCL (pH 6.8), 0.1% glycerol, 10% sodium dodecyl sulfate (SDS), 0.05% 2-β-mercaptoethanol, and bromophenol blue in distilled water) was used to dilute the concentration of quantified protein to 2 mg/mL. After being heated in a boiling water bath for 15 min, 15-μL samples were loaded onto a 10% SDS-polyacrylamide gel and separated using a Mini-Protean system (Bio-Rad, Hercules, CA, USA). The samples were electrotransferred to a polyvinylidene difluoride membrane (Millipore) and blocked with skim milk (BD Biosciences, San Jose, CA, USA). The membrane was incubated with PGP9.5 antibody diluted to 1:500 in skim milk overnight at 4 °C. For the negative control, the membrane was treated with normal mouse immunoglobulin G (IgG) at the same concentration of primary antibody. Horseradish peroxidase-conjugated anti-mouse IgG diluted in skim milk (1:10,000) was used as a secondary antibody.

### 2.3. Immunofluorescence

Immunofluorescence was performed using a previously described protocol [12]. The testicular tissues (5 μm), which were attached to the slides, were then deparaffinized using xylene solution. The tissues were dehydrated with a series of ethanol baths (100%, 95%, 80%, 50%, and 25%), treated in a citrate buffer for 30 min at 97.5 °C for antigen retrieval, and cooled via a cooling process for 30 min to room temperature. The slides were blocked with 5% donkey serum (Sigma, St. Louis, MO, USA) diluted in phosphate-buffered saline. The slides were co-stained with mouse anti-human PGP9.5 antibody and goat anti-human DAZL antibody diluted in blocking buffer at the ratio of 1:1000 and 1:100, respectively. Donkey anti-mouse IgG (Alexa Fluor 488, Thermo Fisher Scientific, Waltham, MA, USA) and donkey anti-goat IgG (Alexa Fluor 594, Thermo Fisher Scientific, Waltham, MA, USA) were used as secondary antibodies.

### 2.4. Imaging

Immunolabeling of the sample tissues was observed using a Leica DM2500 fluorescent microscope (Leica, Wetzlar, Germany). The microscope was equipped with an EL 6000 external light source (Leica). Green and red fluorescent expressions were observed using a dual-emission fluorescein-5-isothiocyanate (FITC)/ tetramethyl rhodamine isothiocyanate (TRITC) filter. Cells showing green or red fluorescence were considered positive for either PGP9.5 or DAZL, whereas cells with no fluorescence were considered negative. The immunolabeled images were captured using a Leica DFC 450 C digital camera.

## 3. Results

### 3.1. Cross-Reactivity of the PGP9.5 Antibody in Donkey Testes

**Figure 1:**
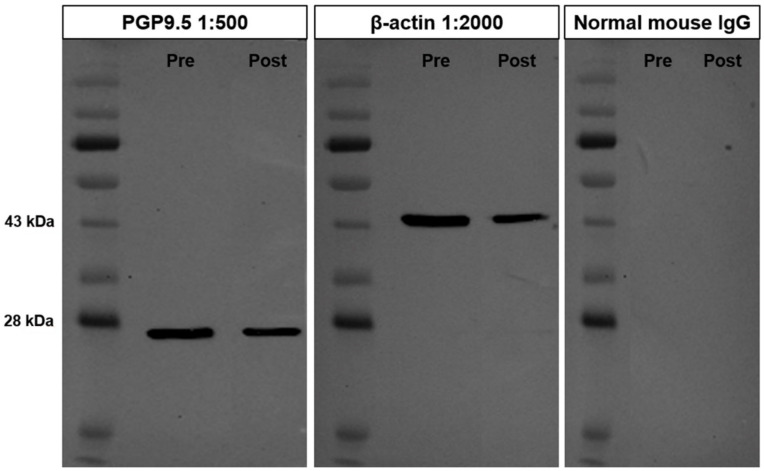
Cross-reactivity of protein gene product (PGP)9.5 in donkey testes. The PGP9.5 protein band appeared at the molecular size of approximately 27 kDa. The β-actin band was also detected at approximately 45 kDa in donkey testes. The negative control lane was treated with normal mouse IgG with the same concentration as the primary antibody and showed no bands.

The cross-reactivity of the PGP9.5 antibody in testicular tissues of pre-pubertal and post-pubertal donkeys was evaluated using Western blot analysis. The protein band appeared in the PGP9.5 antibody at approximately 27 kDa (Figure 1). However, the band was not observed in the negative control lane that was treated with normal mouse IgG with the same concentration as the primary antibody. The β-actin band, which was used for positive control, was also detected at an approximate molecular weight of 45 kDa in donkey testes. This result proves that the mouse anti-human PGP9.5 antibody used in this study suggests cross-reactivity for the PGP9.5 protein of donkey testes.

### 3.2. Identification of Reproductive Stageof Donkey Testes

**Figure 2:**
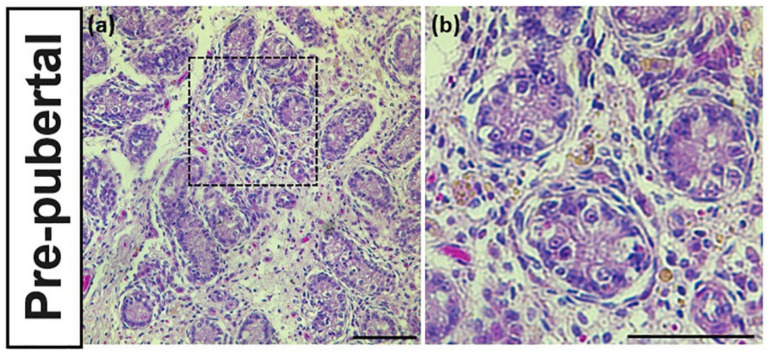

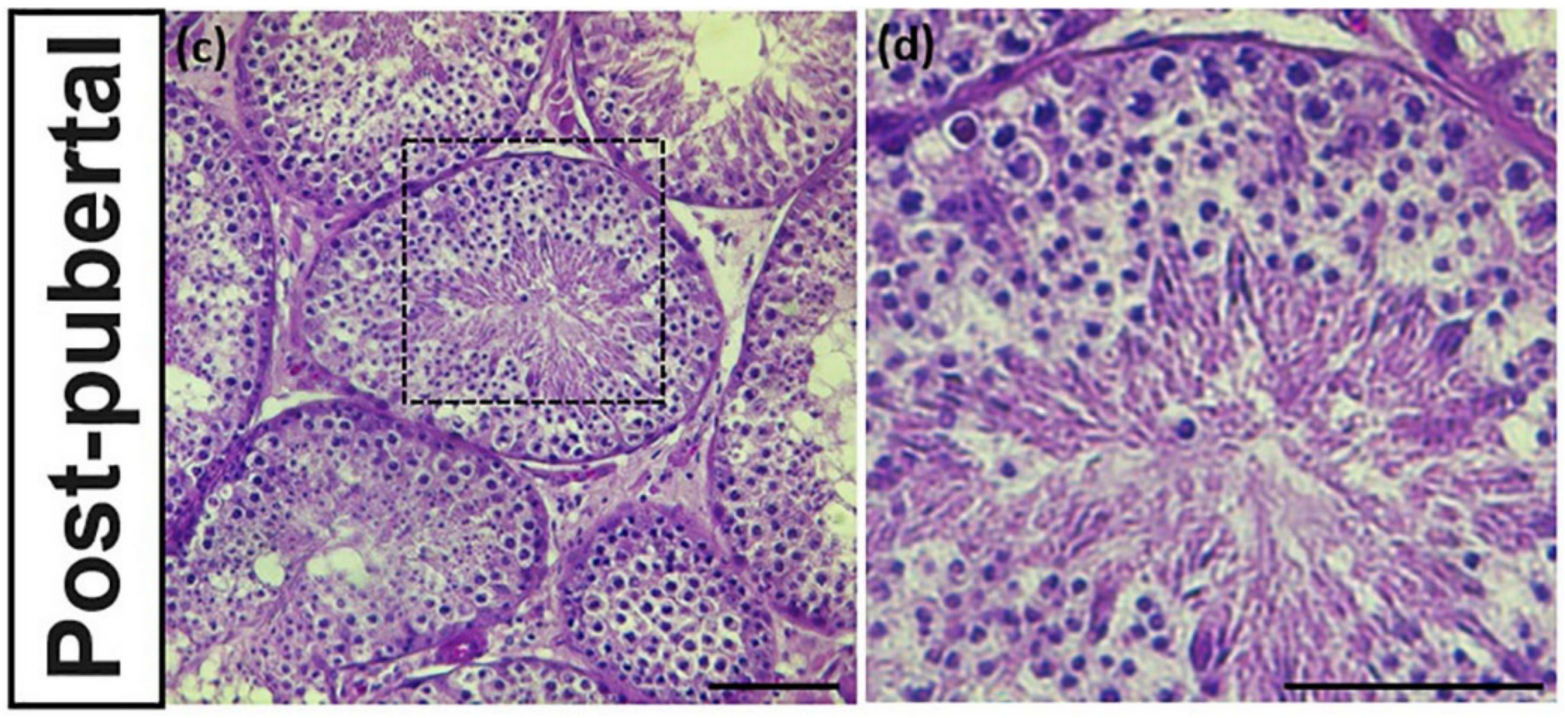
Morphological characteristics of seminiferous tubules of pre- and post-pubertal donkey with H&E staining. Pre-pubertal donkey testes had undeveloped seminiferous tubules with lumen closing (**a**,**b**). In contrast, post-pubertal donkeys had all developmental stage of germ cells with lumen opening gin the seminiferous tubules (**c,d**). The size bar = 50 μm.

The reproductive stage of donkeys is characterized according to the seminiferous tubular morphology. The testes sample of pre-pubertal donkeys had relatively undeveloped seminiferous tubules with lumen closing (Figure 2a,b). In contrast, the seminiferous tubules of post-pubertal donkeys had all developmental stage of germ cells with lumen opening (Figure 2c,d).

### 3.3. PGP9.5 and DAZL Expression in the Testes of Pre-Pubertal Donkeys

**Figure 3:**
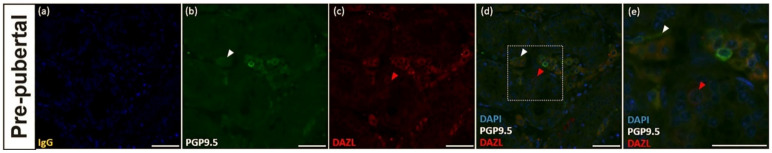
Immunostaining of PGP9.5 and deleted in azoospermia-like (DAZL) in the seminiferous tubules of pre-pubertal donkeys. Testicular tissue stained with IgG showed no immunolabeling (**a**). PGP9.5 immunolabeling was detected in the cytoplasms of myoid cells (**b**,**d**,**e**). The DAZL immunolabeling was detected in the cytoplasm of germ cells (**c**,**d**,**e**). The white arrowhead indicates myoid cells immunolabeled with PGP9.5. The red arrowhead indicates germ cells immunolabeled with DAZL. The size bar = 50 μm.

The deleted in azoospermia-like (DAZL) is a molecular marker for undifferentiated spermatogonia of pre-pubertal donkeys [10]. In this study, the expression of DAZL was detected in the spermatogonia in pre-pubertal testicular tissues (Figure 3c,d,e). The immunolabeling of PGP9.5 in testicular tissue was not observed in germ cells in the seminiferous tubules. However, the cytoplasms of myoid cells located basal lamina in seminiferous tubules were immunostained with PGP9.5 (Figure 3b,d,e).

### 3.4. PGP9.5 and DAZL Expressions in Testes of Post-Pubertal Donkeys in Different Seminiferous Epithelium Cycle Stages 

**Figure 4:**
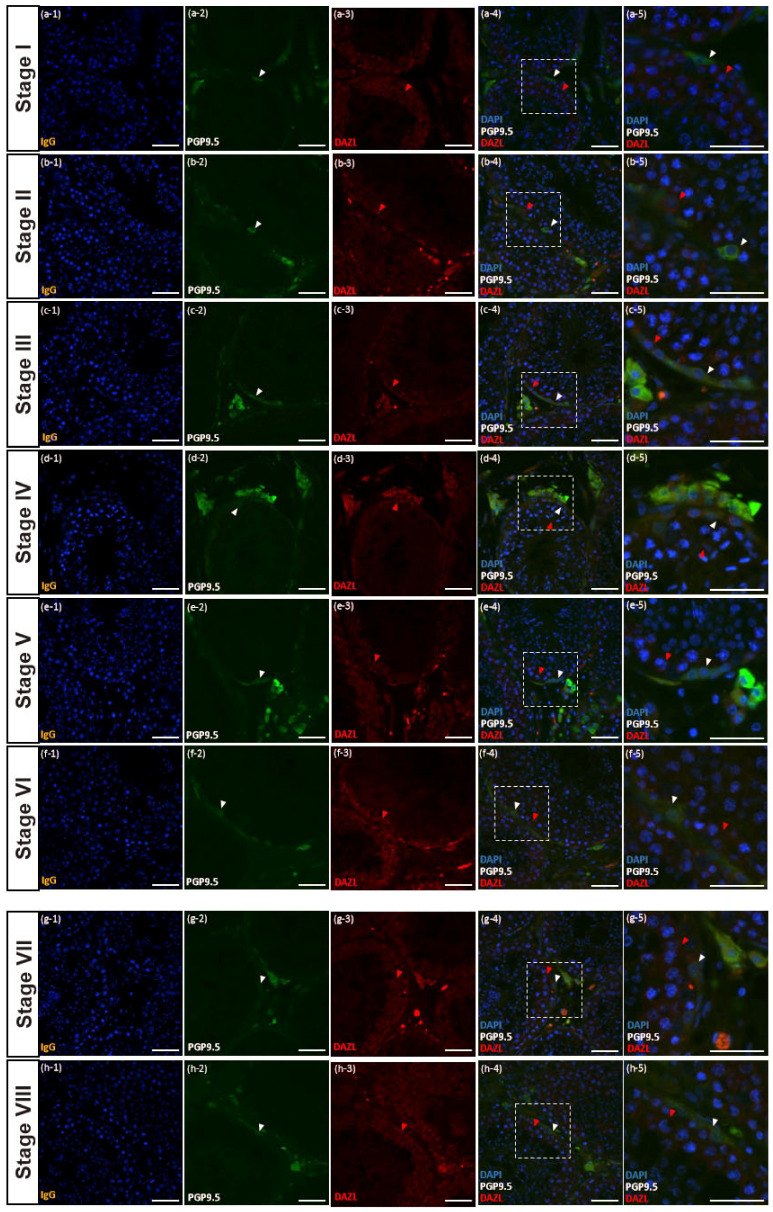
Reproductive stage-dependent expression of PGP9.5 and DAZL in the spermatogonia of a post-pubertal donkey. PGP9.5 immunolabeling was discovered in the cytoplasm of spermatogonia in the post-pubertal stage co-stained with DAZL (**a**–**h**). The white arrowhead indicates germ cells immunolabeled with PGP9.5. The red arrowhead indicates germ cells immunolabeled with DAZL. The size bar = 50 μm.

The stages of the seminiferous epithelium cycle of donkey testes were categorized with eight stages following the results of previous studies [13,14] to determine the germ cell types immunostained with PGP9.5. The DAZL is a molecular marker of undifferentiated spermatogonia and primary spermatocytes in the post-pubertal stage of donkeys [10]. Most spermatogonia and primary spermatocytes near the basal membrane of seminiferous tubules were immunostained with DAZL antibody in all reproductive stages (Figure 4a(3–5), b(3–5), c(3–5), d(3–5), e(3–5), f(3–5), g(3–5), and h(3–5)). In addition, PGP9.5 displayed positive expression in spermatogonia in all eight different stages of the seminiferous epithelium cycle (Figure 4a(4–5), b(4–5), c(4–5), d(4–5), e(4–5), f(4–5), g(4–5), and h(4–5)). The PGP9.5 was co-immunolabeled with DAZL from stages 3 to 8 (c(4–5), d(4–5), e(4–5), f(4–5), g(4–5), and h(4–5)), whereas PGP9.5 and DAZL were immunolabeled, respectively, in spermatogonia in stages 1 and 2 (Figure 4a(4–5) and b(4–5)).

### 3.5. Expression of PGP9.5 in Leydig Cells of the Testes of Donkeys

**Figure 5:**
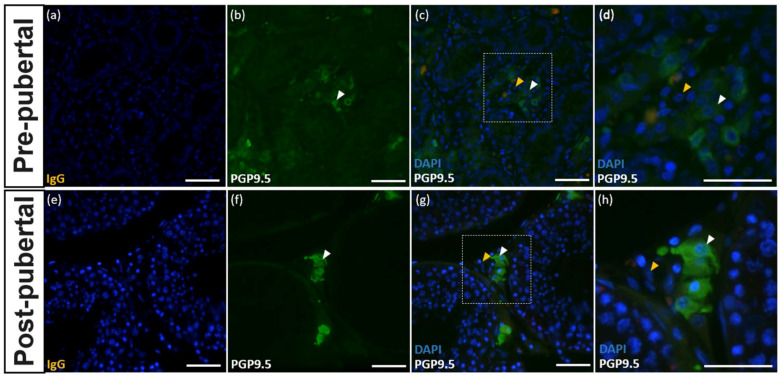
Expression of PGP9.5 in Leydig cells of pre- and post-pubertal donkeys. Testicular tissue stained with IgG showed no immunolabeling (**a**,**e**). The expression of PGP9.5 was observed in the testes of pre-pubertal (**b**–**d**) and post-pubertal (**f**–**h**) donkeys. The white arrowheads indicate Leydig cells stained with PGP9.5. The yellow arrowheads indicate Leydig cells without PGP9.5 staining. The white bar = 50 μm.

Leydig cells were immunolabeled with the PGP9.5 antibody at both the pre-pubertal and post-pubertal stages (Figure 5a–h). At both reproductive stages, PGP9.5 expression was localized in the cytoplasm of Leydig cells.

## 4. Discussion

This study was conducted to find molecular markers to specify germ cell developmental stages in donkey testes. First, we demonstrated the cross-reactivity of each antibody to its respective protein in the testicular tissues of donkeys using Western blot analysis. The PGP9.5 protein band appeared at the adequate molecular size (~27 kDa). The antibodies also had cross-reactivity with PGP9.5 expressed in donkey testicular tissues, similar to a result seen in horses [15]. UCHL1 mRNA of horses is 99.44% identical with donkey mRNA based on the sequence analysis. The similar immunolabeling pattern of UCHL1 between horse and donkey testes appears to be due to high rate of sequence similarity. In addition, in the study of chromosomal polymorphisms in the genus *Equus*, authors suggested that the chromosome arms are homologous in UCHL1 [16]. Such results indicate that the structural motifs recognized by the PGP9.5 antibody are identical in the two species.

PGP9.5 is observed in neuroendocrine cells and tumors; it is also used for the immunohistochemical detection of SSCs in several animals such as mice [17], bovines [18], goats [9], and porcine [19]. Since SSCs exist in the testes of either pre-mature or mature males, we expected that the expression of PGP9.5 is present in both the pre- and post-pubertal tissues of donkeys. However, in the pre-pubertal stage, PGP9.5 was not immunolabeled in germ cells, while post-pubertal stage spermatogonia were immunolabeled with the PGP9.5 antibody. This result indicates that PGP9.5 expression in spermatogonia is reproductive stage-dependent in the donkey.

PGP9.5 is an essential modulator for germ cell apoptosis in testes [20,21,22]. The apoptosis of germ cells regulates the number of germ cells by modulating dynamic cell differentiation of spermatogenesis. In addition, apoptosis can eliminate defective germ cells during active spermatogenesis to maintain testicular homeostasis [23,24]. During the pre-pubertal period, the gonocytes are gradually replaced with spermatogonia, which is reserved, rather than undergoing active differentiation [25,26]. The static condition of spermatogenesis during the pre-pubertal stage may be why PGP9.5 expression was not expressed in the seminiferous tubules of pre-pubertal donkeys.

In post-pubertal tissues, seminiferous tubules were categorized with eight different cyclic stages based on the stage of the seminiferous epithelium cycle of the donkey [13,14]. In stages 1 and 2, spermatogonia were immunolabeled with either PGP9.5 or DAZL. In contrast, the PGP9.5 and DAZL were co-immunolabeled in some spermatogonia in stages 3 to 8. The distinct morphological difference of spermatogonia immunolabeled with PGP9.5 only compared to immunolabeled with PGP9.5 and DAZL was the absence of cytoplasmic bridges. Type A SSCs do not have cytoplasmic bridges with which progenitor cells are connected [27]. Thus, spermatogonia immunolabeled with PGP9.5 only should be SSCs in donkeys. We previously reported that DAZL might be a marker for differentiating and differentiated spermatogonia and primary spermatocytes in donkeys [10]. Thus, germ cells stained with DAZL only in stages 1 and 2 appear to be differentiating spermatogonia. In the stages of 3 to 8, several PGP9.5-positive spermatogonia were connected with cytoplasmic bridges and placed adjacent to the membrane of seminiferous tubules. This result also indicates that PGP9.5 is expressed in the cytoplasmic area of spermatogonia A and B.

Interestingly, Leydig cells of donkeys were also immunolabeled with PGP9.5 at both pre- and post-pubertal stages in this study. Our finding is consistent with previous research in other species such as horses [15] and humans [28], showing the strong intensity of immunolabeling in the cytoplasmic area of Leydig cells. However, this result is contrary to the results found in domestic dogs [29] and mice [17]. The results of those studies suggest that PGP9.5 expression in Leydig cells is species-dependent. In horses, all types of Leydig cells were immunolabeled with PGP9.5 [15]. In contrast, in donkey testes, some Leydig cells were immunolabeled with PGP9.5 in both pre- and post-pubertal stages. Based on the developmental stages, different stages of Leydig cells can be identified as stem, progenitor, immature, adult, or aged Leydig cells [30]. In this study, we could not identify the stages of Leydig cells immunolabeled with PGP9.5. Interestingly, in the pre-pubertal stages, various Leydig cells were immunolabeled with PGP9.5, whereas PGP9.5-positive Leydig cells had a round nucleus and wide cytoplasmic area, assuming that Leydig PGP9.5-positive cells are adult Leydig cells. However, further study should be conducted to confirm the developmental stages of Leydig cells expressing PGP9.5 at different reproductive stages. The results of this study indicate that PGP9.5 is a critical molecular marker for the study of donkey Leydig cell development.

## 5. Conclusions

In conclusion, PGP9.5 expresses in germ cells and Leydig cells differently depending on the reproductive stage. PGP9.5 antibody can be used as a marker to identify undifferentiated, differentiating, and differentiated donkey spermatogonia. This antibody can also be used as a marker to study the development of Leydig cells in donkey testes.
